# Supplementary material for: Cataract-Associated New Mutants S175G/H181Q of βΒ2-Crystallin and P24S/S31G of γD-Crystallin Are Involved in Protein Aggregation by Structural Changes
Source: Int J Mol Sci. 2020 Sep 5;21(18):6504. doi: 10.3390/ijms21186504 (PMC7555777; doi:10.3390/ijms21186504)
Supplement: Supplementary file 1 [file ijms-21-06504-s001.pdf]

Supporting Information

Structural Changes of Two Novel Crystallin Mutants Originated from Cataract Patients and its Involvement in Protein Aggregation

*In-Kang Song[1], Seung-Jin Na[2], Eunok Paek[2] Kong-Joo Lee[1]**

[1] Graduate School of Pharmaceutical Sciences and College of Pharmacy, Ewha Womans University, Seoul 03760, South Korea.

[2] Department of Computer Science, Hanyang University, Seoul 04763, South Korea

*Corresponding author : kjl@ewha.ac.kr

Figure S1. Location of each mutation listed in Table 1. in the crystallin structures (PDB entry ID; a:2Y1Y, b:3LWK, c:1OKI, d:1YTQ and e-f:3QK3)

Figure S2. The hydrogen bonds interaction predicted by the Chimera for βB2-crystallin structure (PDB entry ID 1YTQ). Ser175 and His181 are shown in red and Gln183 interacting with His181 is in blue. The solid blue line represents the predicted hydrogen bonds.

Figure S3. Crystal structure of γD-crystallin (PDB entry ID 1HK0). Pro24 and Ser31 are shown in red and Arg15 and 32 are in blue. The distances to Arg15, 32 and Ser31 are shown as dot line, respectively.

Figure S4. Oligomerization pattern of recombinant proteins of WT and S175G/H181Q mutant of βB2-crystallin under oxidative stress. Proteins were separated via SDS-PAGE with or without β-ME and stained with Coomassie blue.

Figure S5. Hela cells were plated on the glass coverslip 24 h before transfection. Cells were then transfected with Flag, Flag βB2-crystallin or Flag γD-crystallin. After 24 h, cells were stained Flag-crystallin (green) and nucleus (blue) under confocal microscopy. Cells were photographed at x 60 magnification. All of the Western blot results were selected representative data from more than duplicated results.

Figure S6. Long-term stability revealed by incubating 5.0 mg/ml proteins at 37°C continuously, and turbidity data were measured at given intervals.

Figure S7. Structure of of γD-crystallin (PDB entry ID 1HK0). Pro24 and Ser31 are shown in red and Cysteines are in blue. A monomeric molecules of γD-crystallin crystal structure by ribbons in left panel. The accessible surface of the γD-crystallin with corresponding amino acid residues in right panel. The surface exposed cysteines are Cys19, 42, 109 and 111.

Figure S8. Representative MS/MS spectra of post-translationally modified peptides of γD-crystallin listed in Table 2. Spectra are in the order in the list.

Figure S9. Sequence alignments of βB2-crystallin (a) and γD-crystallin (b) near mutation.

Figure S10. Composition ratio of identified crystallin proteins in each sample

Table S1. Comparison of differential deuterium exchange rates of identified WT and mutant of βB2-crystallin peptides in HDX-MS experiment.

Table S2. Identified PSMs with a minimum peptide length of 8 were identified at FDR 1%

Table S3. Identified Mutations.

Table S4. The list of possible mutations from nuclear cataract human lens samples. (a) Numbers of mutations observed in each crystallin. A total of 112 mutations were observed in our analyses. (b) Numbers of mutations observed in each sample.

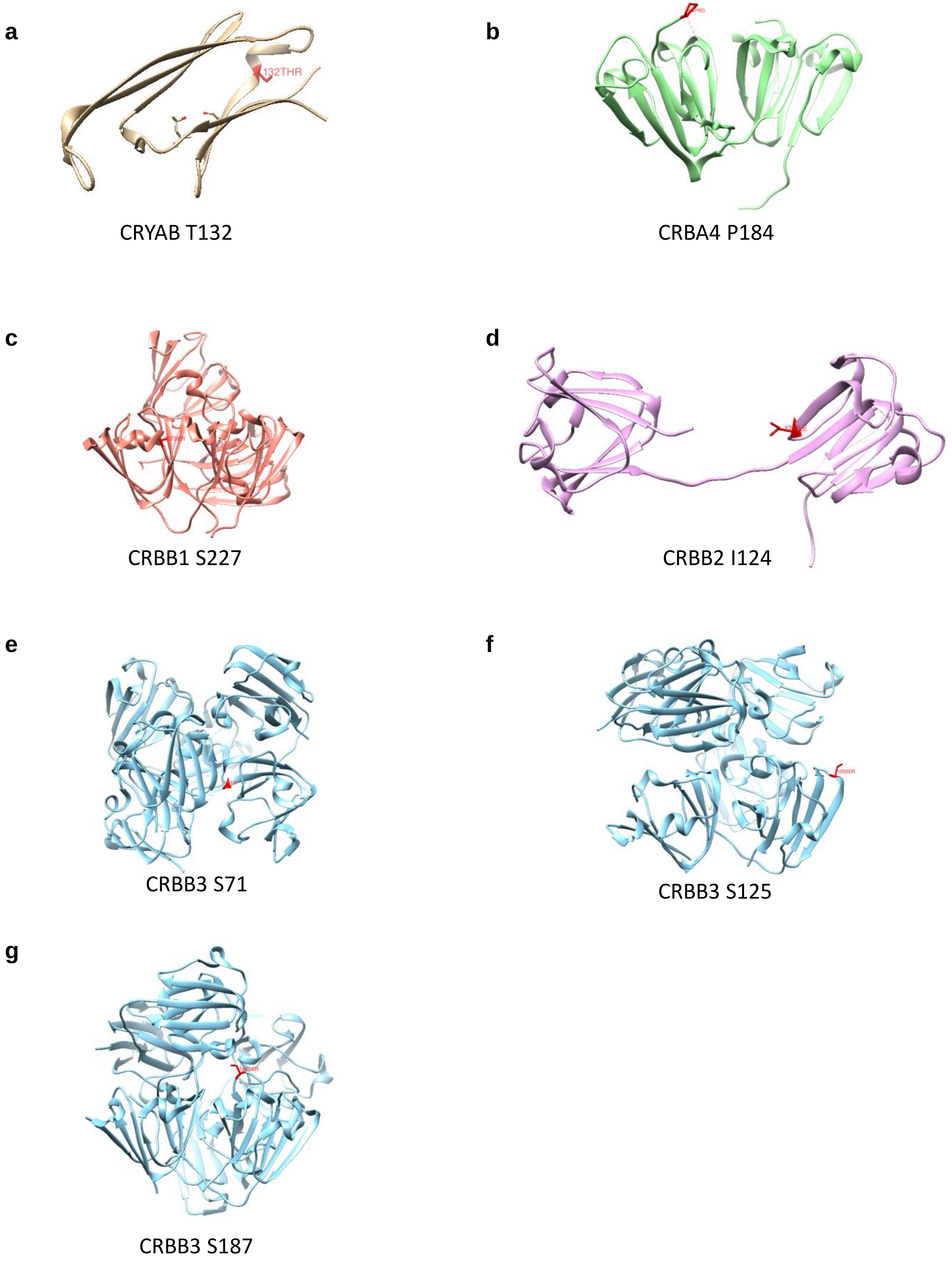

Figure S1. Location of each mutation listed in Table 1. in the crystallin structures (PDB entry ID; a:2Y1Y, b:3LWK, c:1OKI, d:1YTQ and e-g:3QK3)

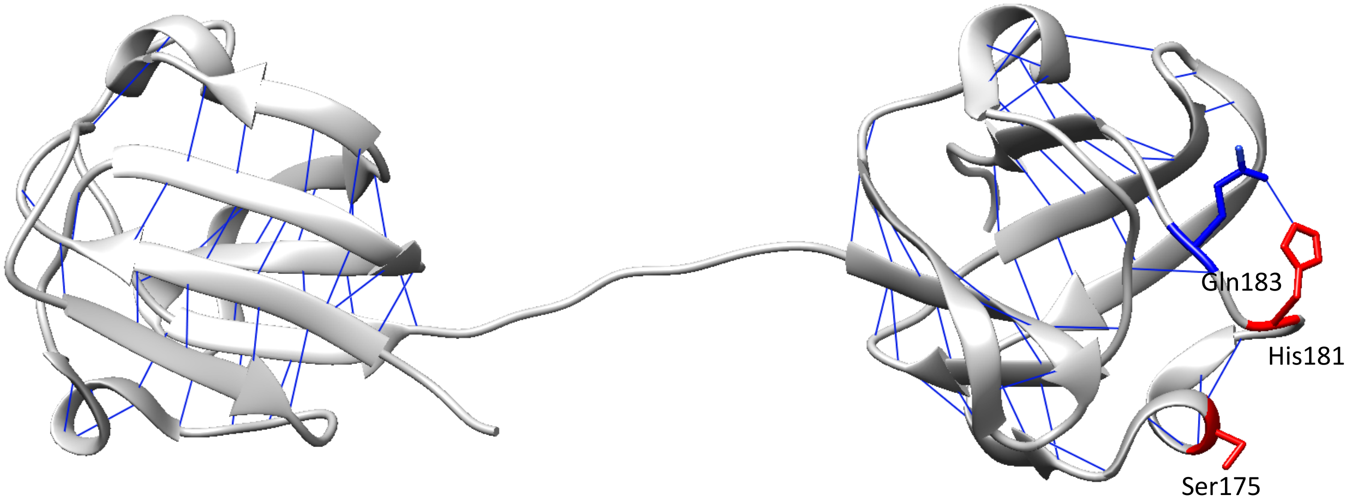

Figure S2. The hydrogen bonds interaction predicted by the Chimera for βB2-crystallin structure (PDB entry ID 1YTQ). Ser175 and His181 are shown in red and Gln183 interacting with His181 is in blue. The solid blue line represents the predicted hydrogen bonds.

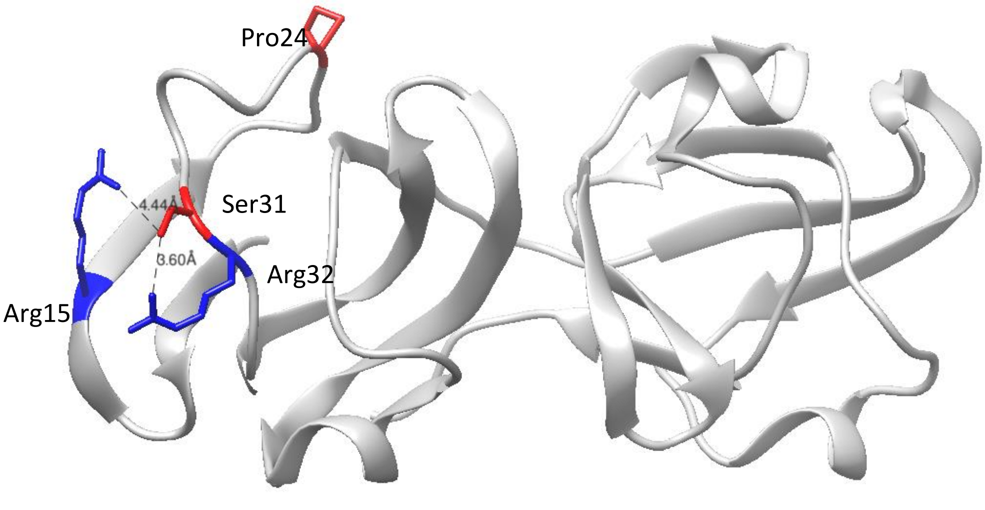

Figure S3. Crystal structure of γD-crystallin (PDB entry ID 1HK0). Pro24 and Ser31 are shown in red and Arg15 and 32 are in blue. The distances to Arg15, 32 and Ser31 are shown as dot line, respectively.

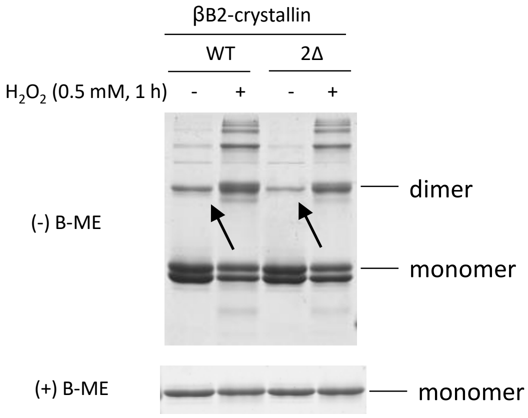

Figure S4. Oligomerization pattern of recombinant proteins of WT and S175G/H181Q mutant of βB2-crystallin under oxidative stress. Proteins were separated via SDS-PAGE with or without β-ME and stained with Coomassie blue.

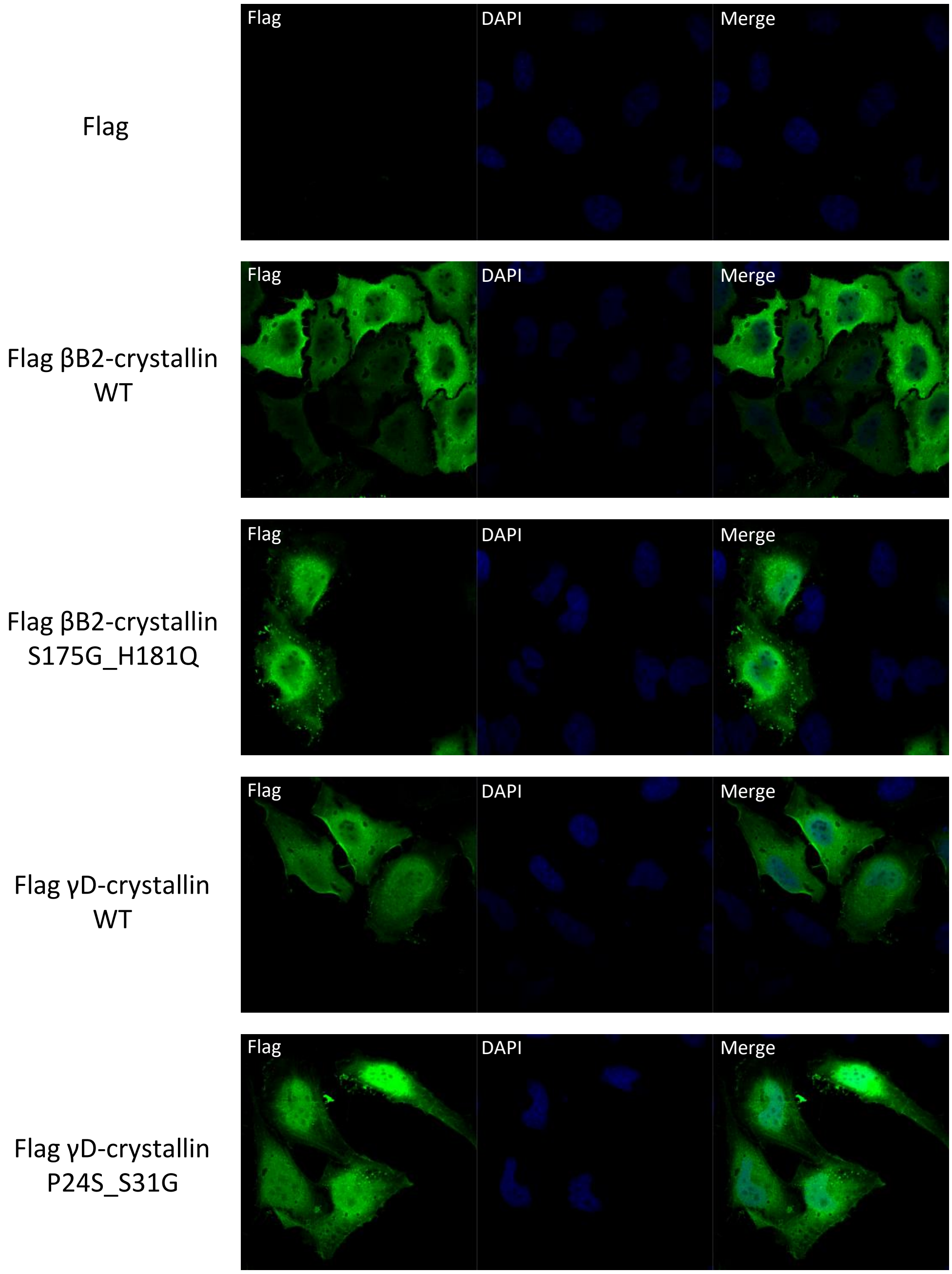

Figure S5. Hela cells were plated on the glass coverslip 24 h before transfection. Cells were then transfected with Flag, Flag βB2-crystallin or Flag γD-crystallin. After 24 h, cells were stained Flag-crystallin (green) and nucleus (blue) under confocal microscopy. Cells were photographed at x 60 magnification. All of the Western blot results were selected representative data from more than duplicated results.

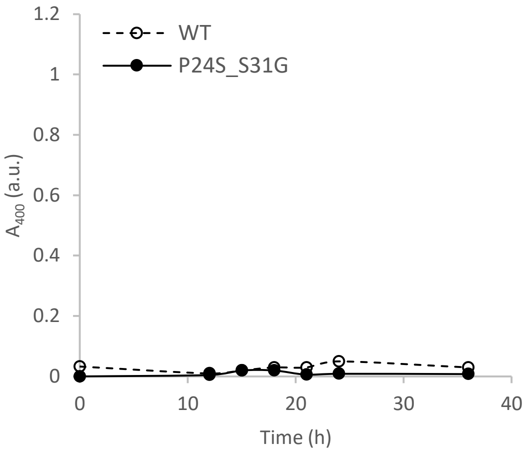

Figure S6. Long-term stability revealed by incubating 5.0 mg/ml proteins at 37°C continuously, and turbidity data were measured at given intervals.

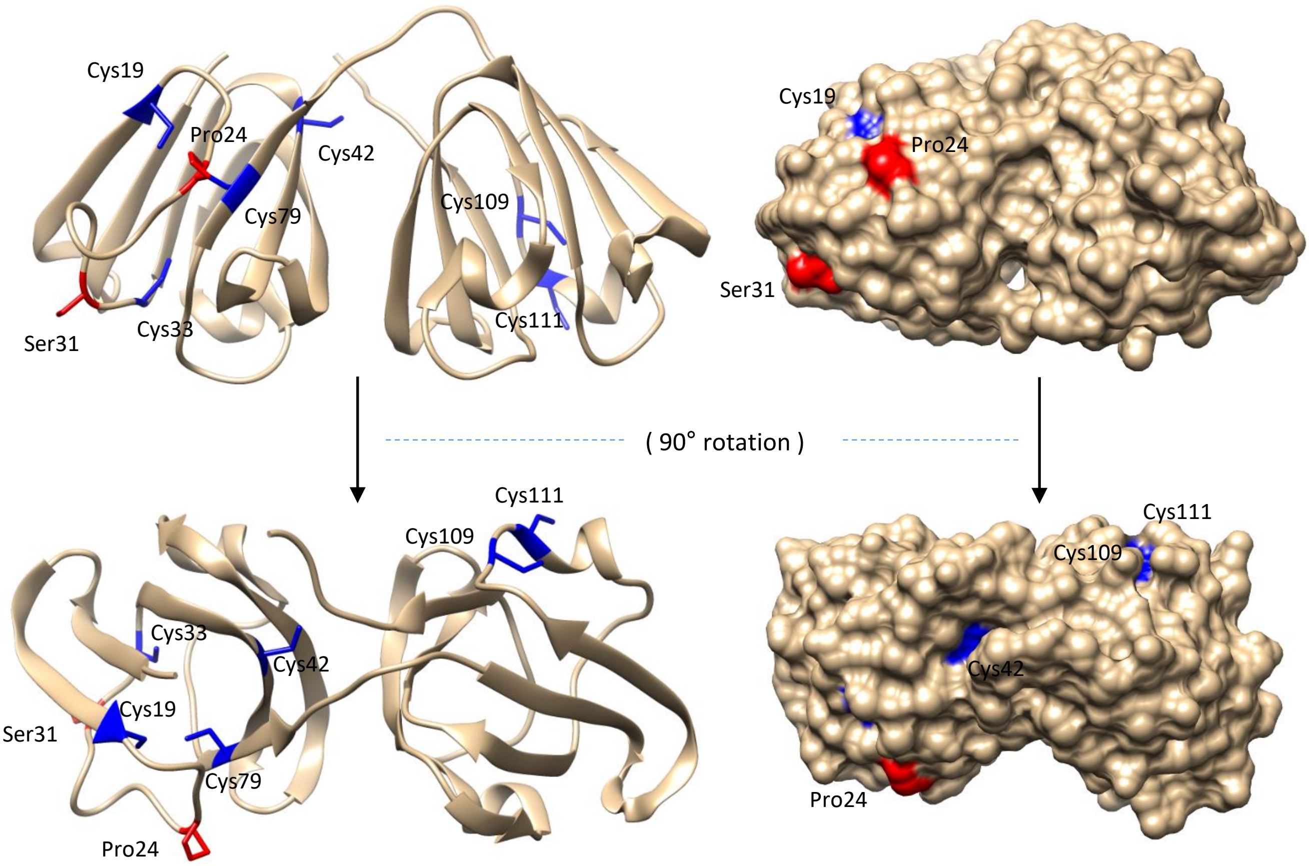

Figure S7. Structure of of γD-crystallin (PDB entry ID 1HK0). Pro24 and Ser31 are shown in red and Cysteines are in blue. A monomeric molecules of γD-crystallin crystal structure by ribbons in left panel. The accessible surface of the γD-crystallin with corresponding amino acid residues in right panel. The surface exposed cysteines are Cys19, 42, 109 and 111.

^{16}HYECSSDHPNLQPYLSR^{32} + Dioxidation(C19)

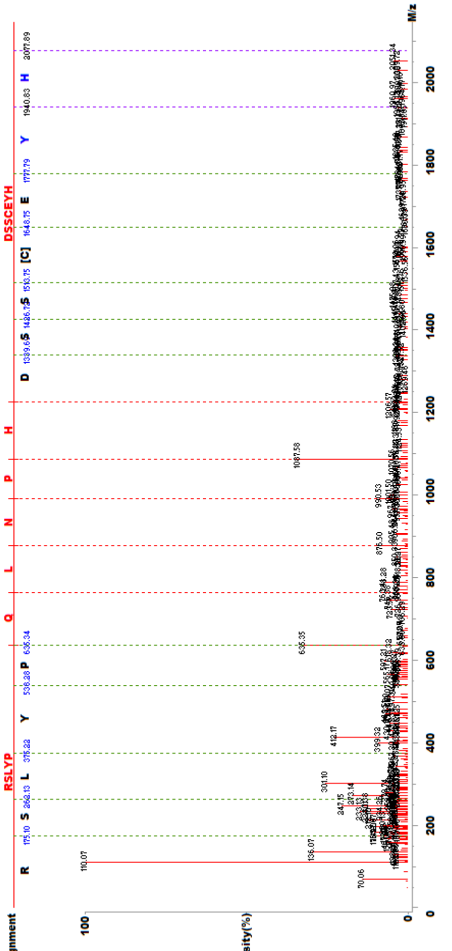

^{16}HYECSSDHPNLQPYLSR^{32} + NEM (C19)

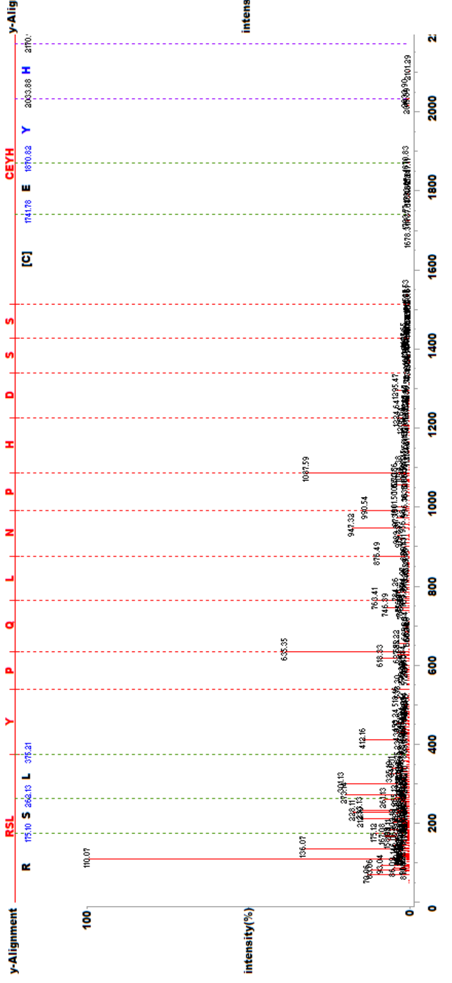

^{38}VDSGCWMLYEQPNYSGLQYFLR^{59} + thiosulfinate (C42)

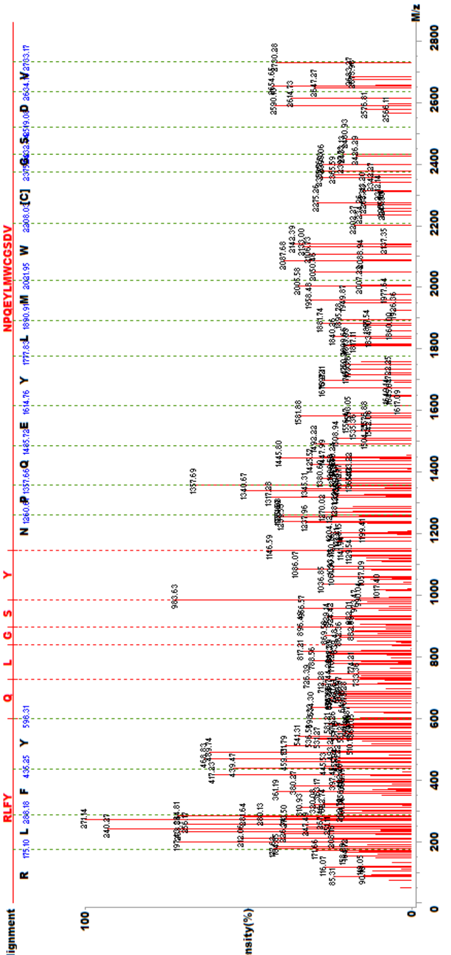

^{38}VDSGCWMLYEQPNYSGLQYFLR^{59} + NEM (C42)

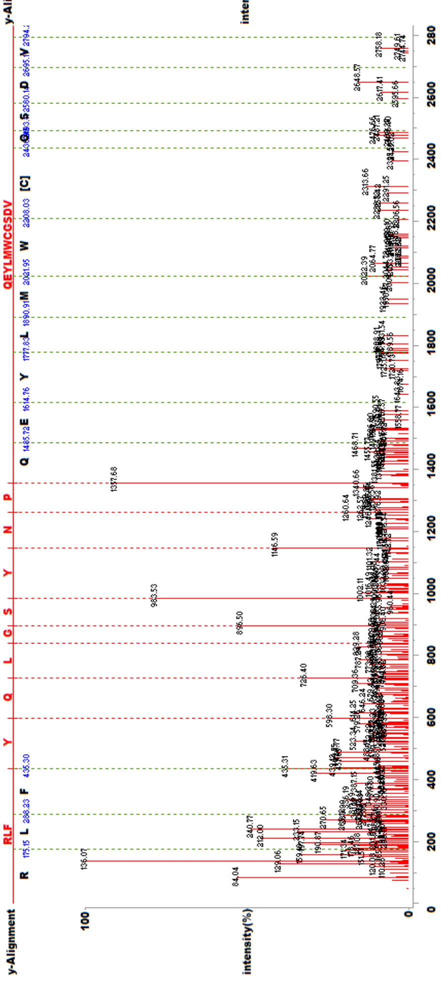

Figure S8. Representative MS/MS spectra of post-translationally modified peptides of γD-crystallin listed in Table 2. Spectra are in the order in the list.

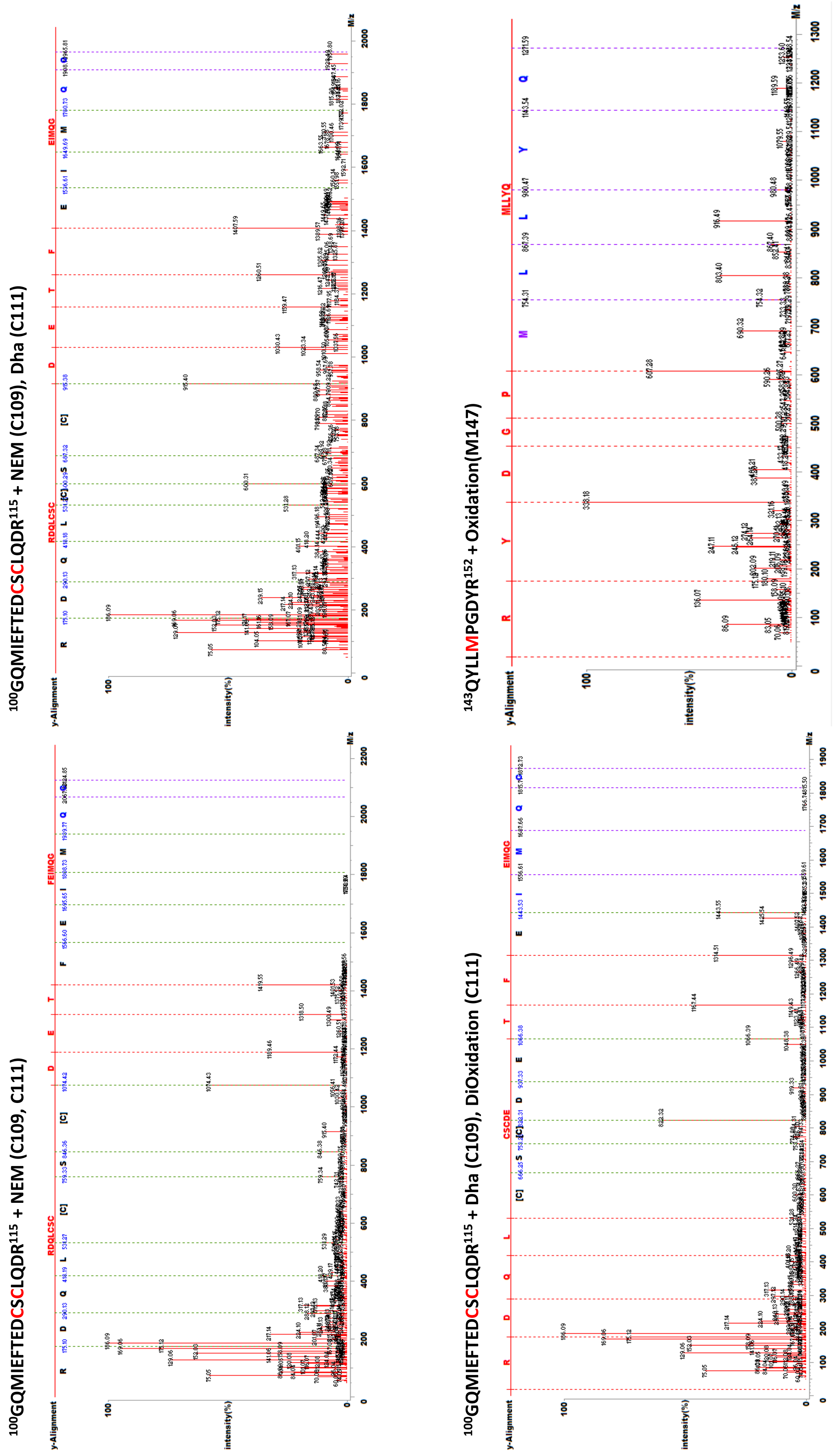

Figure S8. (Continued)

^{143}QYLLMPGDYR^{152} + Dioxidation(M147)

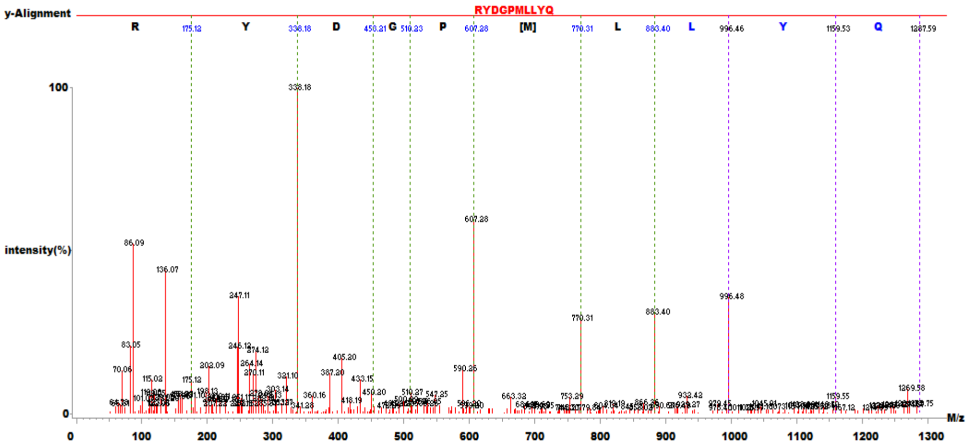

Figure S8. (Continued)

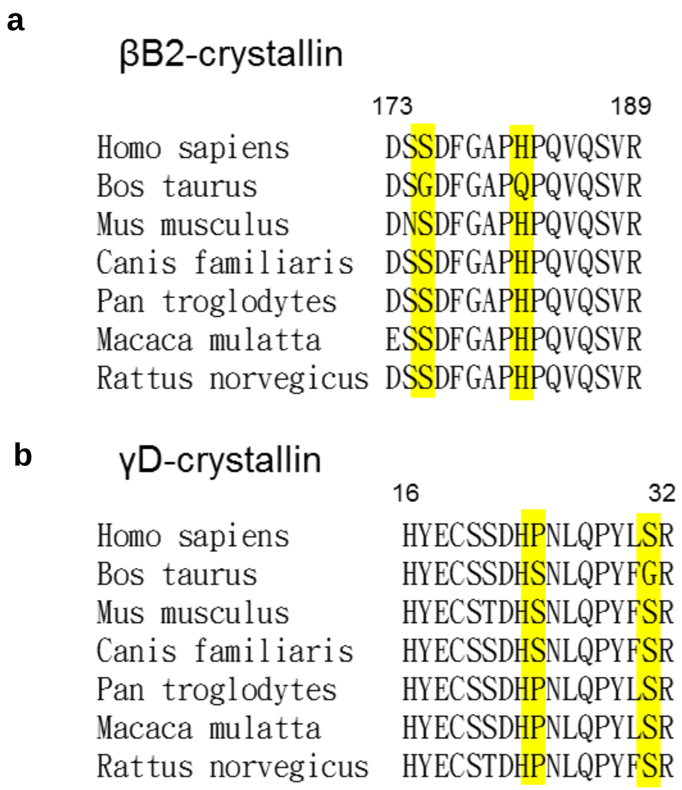

Figure S9. Sequence alignments of βB2-crystallin (a) and γD-crystallin (b) near mutation.

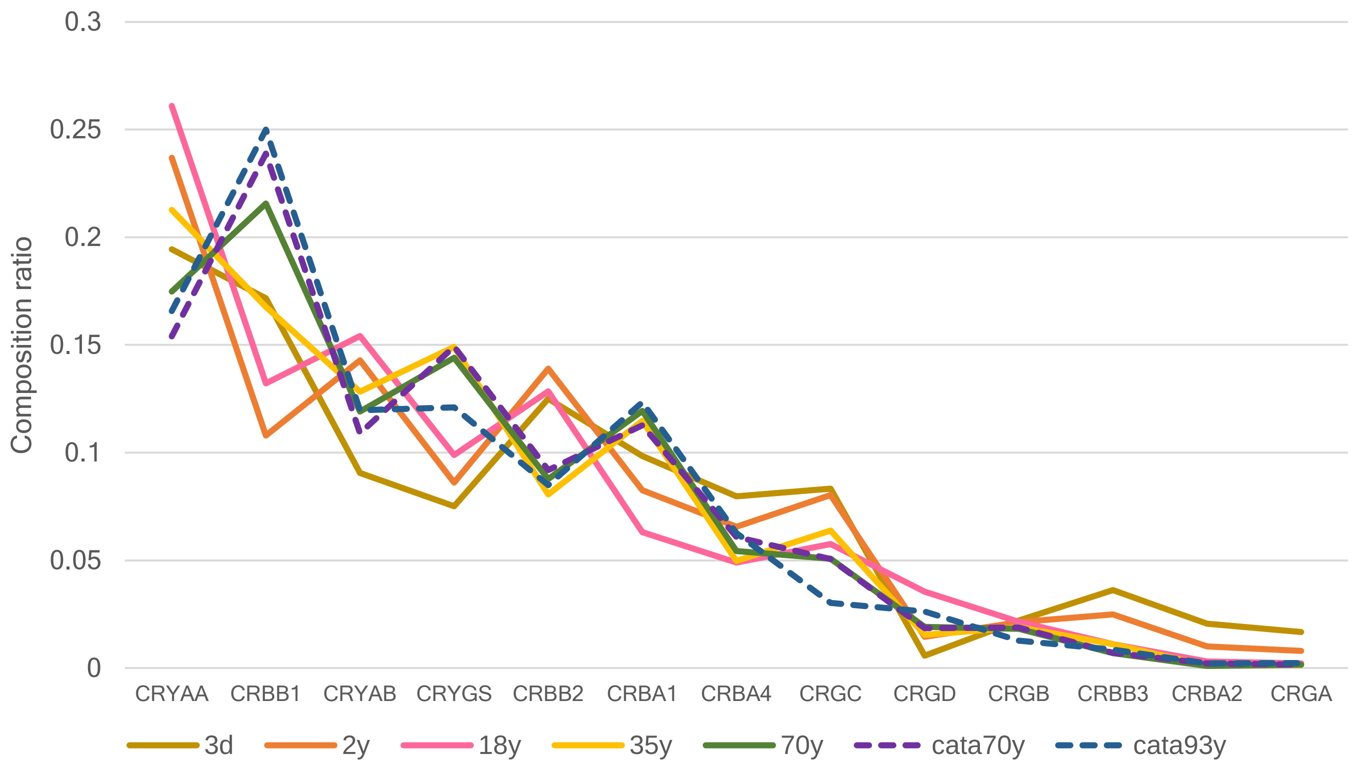

Figure S10. Composition ratio of identified crystallin proteins in each sample

Table S1. Comparison of differential deuterium exchange rates of identified WT and mutant of βB2-crystallin peptides in HDX-MS experiment.

Sequence	Start	End	Region	Domain	Differential deuterium exchange rate (%)				Mean
					10 sec	60 sec	300 sec	9000 sec	
MASDHQTQAGKPQSLNPKI	1	19	N-terminal arm		0.05	0.05	0.05	0.00	0.04
MASDHQTQAGKPQSLNPKIIIF	1	22			0.00	-0.05	0.00	-0.05	-0.02
MASDHQTQAGKPQSLNPKIIIFE	1	23			0.00	0.00	0.00	0.00	0.00
AG KPQSLNPKIIIF	9	22		Greek key 1	0.07	0.00	0.00	0.00	0.02
EQENFQGHSHELNGPCPNL	23	41			0.00	0.00	0.00	0.00	0.00
EQENFQGHSHELNGPCPNLKETGVE	23	47			0.00	0.00	0.00	0.00	0.00
EQENFQGHSHELNGPCPNLKETGVEKAGSVL	23	53			0.03	-0.03	0.00	-0.03	-0.01
NFQGHSHELNGPCPNL	26	41			0.00	0.06	0.00	0.00	0.02
NFQGHSHELNGPCPNLKETGVEKAGSVL	26	53			0.00	0.04	0.04	0.00	0.02
KETGVEKAGSVL	42	53			0.00	0.00	0.00	0.00	0.00
VQAGPWVGYEQA	54	65			0.00	0.00	-0.08	0.00	-0.02
VQAGPWVGYEQANCKGEQ	54	71			0.00	0.06	-0.06	-0.06	-0.01
VQAGPWVGYEQANCKGEQF	54	72			0.00	0.00	0.00	0.00	0.00
YEQANCKGEQF	62	72		Greek key 2	-0.09	0.00	-0.09	0.00	-0.05
FVFEKGEYPRW	72	82			0.00	0.00	0.00	0.00	0.00
FVFEKGEYPRWDS	72	84			0.08	0.00	0.00	0.00	0.02
FVFEKGEYPRWDSW	72	85			0.00	0.00	0.00	-0.07	-0.02
FVFEKGEYPRWDSWTSSRRTDSL	72	94			0.04	-0.04	-0.04	-0.09	-0.03
VFEKGEYPRWDSW	73	85			0.00	0.00	0.00	0.00	0.00
VFEKGEYPRWDSWTSSRRTDSLSSL	73	97			0.00	-0.08	0.00	0.00	-0.02
FEKGEYPRWDSWTSSRRTDSL	74	94			0.05	0.00	0.00	0.00	0.01
SSLRPIKVDSQE	95	106	connecting peptide		0.08	0.08	0.00	0.00	0.04
SSLRPIKVDSQEHKIIL	95	111			0.06	0.00	0.00	0.00	0.01
RPIKVDSQEHKIIL	98	111		Greek key 3	0.00	0.00	0.00	0.00	0.00
YENPNFTGKKMEIID	112	126			0.00	0.00	0.00	0.00	0.00
YENPNFTGKKMEIIDD	112	127			0.00	0.00	0.00	-0.06	-0.02
YENPNFTGKKMEIIDDDVPSF	112	132			0.05	-0.05	0.05	0.00	0.01
YENPNFTGKKMEIIDDDVPSFHAHGYQE	112	139			0.04	0.04	0.00	-0.04	0.01
YENPNFTGKKMEIIDDDVPSFHAHGYQEKVSS	112	143			0.00	-0.03	0.00	0.00	-0.01
TGK KMEIIDDDVPSFHAHGYQEKVSS	118	143			0.00	0.00	0.08	0.00	0.02
DDVPSFHAHGYQEKVSS	127	143			0.06	0.00	0.00	0.00	0.01
HAHGYQEKVSSVRVQSGT	133	150			0.06	0.06	0.00	0.00	0.03
VRVQSGTW	144	151			0.00	0.13	0.13	0.00	0.06
VRVQSGTWVG	144	153			0.00	0.10	0.10	0.00	0.05
VRVQSGTWVGYQYPGYRGLQ	144	163		Greek key 4	0.00	0.05	0.00	0.00	0.01
VRVQSGTWVGYQYPGYRGLQY	144	164			0.05	0.05	0.05	0.00	0.04
VRVQSGTWVGYQYPGYRGLQL	144	165			0.00	0.05	0.00	0.00	0.01
WVGYQYPGYRGLQYL	151	165			0.00	0.07	0.00	0.00	0.02
VGYQYPGYRGLQYL	152	165			0.00	0.07	0.00	0.00	0.02
YQYPGYRGLQ	154	163			0.00	0.00	0.00	-0.10	-0.03
LEKGDYKDSSDFGAPHPQVQS	166	186			0.10	0.05	0.05	0.05	0.06
VRRIRDMQWHQRGAFHPSN	187	205	C-terminal arm		0.05	0.00	0.00	0.00	0.01
MQWHQRGAFHPSN	193	205			0.08	0.08	0.00	0.00	0.04

Table S2. Dataset size and identified PSMs

(*The amount of water-insoluble material from the 3-day old lens was negligible and not analyzed.)

**Human Lens Tissues**		**Sample size (#Spectra)**			**#Identified PSMs at 1% FDR**		
**age**	**condition**	**water-soluble**	**water-insoluble**	**total**	**water-soluble**	**water-insoluble**	**total**
3d	Normal	54,385	0	54,385	6,279	0	6,279
2y	Normal	53,945	56,620	110,565	7,145	8,386	15,531
18y	Normal	56,393	67,847	124,240	5,815	7,978	13,793
35y	Normal	52,286	60,445	112,731	6,279	9,183	15,462
70y	Normal	65,263	64,112	129,375	8,190	7,167	15,357
70y	Cataract	62,325	59,605	121,930	7,154	6,276	13,430
93y	Cataract	65,082	65,283	130,365	7,191	5,730	12,921

Table S3. Identified Mutations.

Protein	Site	AA	Mutation	3d	2y	18y	35y	70y	cat70y	cat93y	#Normal sample	#Cataract sample	Type
sp|P02489|CRYAA_HUMAN	1	M	R	0	0	0	1	0	0	0	1	0	
sp|P02489|CRYAA_HUMAN	12	R	D	0	0	0	0	1	0	0	1	0	
sp|P02489|CRYAA_HUMAN	13	T	G	0	0	0	1	0	1	1	1	2	
sp|P02489|CRYAA_HUMAN	34	Y	W	0	0	0	0	1	1	1	1	2	
sp|P02489|CRYAA_HUMAN	54	R	C	1	0	0	0	0	0	0	1	0	
sp|P02489|CRYAA_HUMAN	59	S	C	0	0	0	2	2	1	0	2	1	
sp|P02489|CRYAA_HUMAN	82	P	S	1	0	0	0	0	0	0	1	0	
sp|P02489|CRYAA_HUMAN	89	V	R	1	0	0	0	0	0	0	1	0	
sp|P02489|CRYAA_HUMAN	98	G	V	0	0	1	0	0	0	0	1	0	
sp|P02489|CRYAA_HUMAN	98	G	T	0	0	0	1	0	0	0	1	0	
sp|P02489|CRYAA_HUMAN	120	L	W	0	0	0	1	0	0	0	1	0	
sp|P02489|CRYAA_HUMAN	129	L	N	0	0	1	0	0	0	0	1	0	
sp|P02489|CRYAA_HUMAN	142	C	S	0	0	0	2	0	0	0	1	0	
sp|P02489|CRYAA_HUMAN	144	P	C	0	0	1	0	0	0	0	1	0	
sp|P02489|CRYAA_HUMAN	156	E	Y	0	0	0	0	0	0	1	0	1	
sp|P02511|CRYAB_HUMAN	57	A	T	0	0	0	0	1	0	0	1	0	
sp|P02511|CRYAB_HUMAN	57	A	R	0	0	0	0	1	0	0	1	0	
sp|P02511|CRYAB_HUMAN	61	F	IL	0	0	0	0	0	1	2	0	2	Cataract-specific
sp|P02511|CRYAB_HUMAN	90	K	D	0	0	0	0	1	0	0	1	0	
sp|P02511|CRYAB_HUMAN	98	I	H	0	3	0	0	0	0	0	1	0	
sp|P02511|CRYAB_HUMAN	124	I	D	0	0	0	1	0	1	0	1	1	
sp|P02511|CRYAB_HUMAN	128	V	T	0	1	0	0	0	0	0	1	0	
sp|P02511|CRYAB_HUMAN	132	T	A	0	0	0	0	0	1	2	0	2	Cataract-specific
sp|P02511|CRYAB_HUMAN	140	D	P	0	1	0	0	0	0	0	1	0	
sp|P02511|CRYAB_HUMAN	146	N	P	0	0	0	1	0	1	0	1	1	
sp|P02511|CRYAB_HUMAN	166	K	E	0	0	1	0	0	0	0	1	0	
sp|P02511|CRYAB_HUMAN	175	K	W	0	0	1	0	0	0	0	1	0	
sp|P05813|CRBA1_HUMAN	97	D	Q	0	0	0	0	0	1	0	0	1	
sp|P05813|CRBA1_HUMAN	165	S	C	0	0	0	0	0	0	3	0	1	
sp|P05813|CRBA1_HUMAN	202	A	G	0	0	0	0	0	1	0	0	1	
sp|P05813|CRBA1_HUMAN	206	Q	R	0	0	0	1	0	0	0	1	0	
sp|P07315|CRGC_HUMAN	1	M	D	0	0	0	0	0	0	1	0	1	
sp|P07315|CRGC_HUMAN	62	E	D	0	0	0	0	0	2	0	0	1	
sp|P07315|CRGC_HUMAN	73	S	N	0	0	0	0	0	1	0	0	1	
sp|P07315|CRGC_HUMAN	73	S	D	0	0	0	0	0	1	0	0	1	
sp|P07315|CRGC_HUMAN	99	K	S	0	0	1	0	0	0	0	1	0	
sp|P07315|CRGC_HUMAN	102	M	IL	0	1	0	0	0	0	0	1	0	
sp|P07315|CRGC_HUMAN	103	M	IL	0	0	1	0	0	0	0	1	0	
sp|P07315|CRGC_HUMAN	110	P	C	0	0	1	0	0	0	0	1	0	
sp|P07315|CRGC_HUMAN	149	Q	G	0	0	0	0	1	0	0	1	0	
sp|P07316|CRGB_HUMAN	1	M	D	0	0	0	0	0	0	1	0	1	
sp|P07316|CRGB_HUMAN	62	E	D	0	0	0	0	0	2	0	0	1	
sp|P07316|CRGB_HUMAN	73	S	D	0	0	0	0	0	1	0	0	1	
sp|P07316|CRGB_HUMAN	73	S	N	0	0	0	0	0	1	0	0	1	
sp|P07316|CRGB_HUMAN	150	G	Q	0	0	0	0	0	1	0	0	1	
sp|P07316|CRGB_HUMAN	150	G	K	1	0	0	1	0	1	0	2	1	
sp|P07316|CRGB_HUMAN	155	F	Y	0	0	0	0	0	0	1	0	1	
sp|P07316|CRGB_HUMAN	161	P	M	0	0	0	0	0	0	1	0	1	
sp|P07320|CRGD_HUMAN	24	P	S	0	0	0	0	0	2	1	0	2	Cataract-specific
sp|P07320|CRGD_HUMAN	31	S	G	0	0	0	0	0	2	1	0	2	Cataract-specific
sp|P07320|CRGD_HUMAN	46	Y	W	0	0	1	0	0	0	0	1	0	
sp|P07320|CRGD_HUMAN	70	M	F	0	0	1	0	0	0	0	1	0	
sp|P07320|CRGD_HUMAN	155	Q	H	0	0	0	0	0	0	1	0	1	
sp|P11844|CRGA_HUMAN	1	M	D	0	0	0	0	0	0	1	0	1	
sp|P11844|CRGA_HUMAN	4	I	R	0	0	0	0	0	0	1	0	1	
sp|P11844|CRGA_HUMAN	10	R	E	0	0	0	0	1	0	0	1	0	
sp|P11844|CRGA_HUMAN	10	R	K	0	0	0	0	0	0	2	0	1	
sp|P11844|CRGA_HUMAN	10	R	W	0	0	0	0	0	0	1	0	1	
sp|P11844|CRGA_HUMAN	10	R	Q	0	0	0	0	2	3	5	1	2	
sp|P22914|CRYGS_HUMAN	3	K	G	0	0	0	0	1	0	0	1	0	
sp|P22914|CRYGS_HUMAN	7	K	P	0	0	0	0	0	2	0	0	1	
sp|P22914|CRYGS_HUMAN	8	I	N	0	0	0	0	1	0	0	1	0	

Table S3. (Continued)

Protein	Site	AA	Mutation	3d	2y	18y	35y	70y	cat70y	cat93y	#Normal sample	#Cataract sample	Type
sp|P22914|CRYGS_HUMAN	14	K	W	0	0	0	0	1	0	0	1	0	
sp|P22914|CRYGS_HUMAN	26	D	C	0	0	0	0	1	0	0	1	0	
sp|P22914|CRYGS_HUMAN	52	R	D	0	0	0	0	0	0	1	0	1	
sp|P22914|CRYGS_HUMAN	111	T	M	0	1	0	0	0	0	0	1	0	
sp|P22914|CRYGS_HUMAN	138	I	Q	0	0	0	0	1	0	0	1	0	
sp|P22914|CRYGS_HUMAN	158	R	D	0	0	0	2	1	2	0	2	1	
sp|P22914|CRYGS_HUMAN	161	I	G	0	0	0	0	1	0	0	1	0	
sp|P22914|CRYGS_HUMAN	165	A	W	0	0	0	1	0	0	0	1	0	
sp|P22914|CRYGS_HUMAN	171	Q	P	0	0	1	1	0	0	0	2	0	
sp|P22914|CRYGS_HUMAN	174	R	M	0	1	0	0	0	0	0	1	0	
sp|P26998|CRBB3_HUMAN	71	S	R	0	0	0	0	0	1	1	0	2	Cataract-specific
sp|P26998|CRBB3_HUMAN	113	H	D	1	2	2	0	0	1	1	3	2	
sp|P26998|CRBB3_HUMAN	125	S	G	0	0	0	0	0	3	5	0	2	Cataract-specific
sp|P43320|CRBB2_HUMAN	32	H	C	0	0	1	0	0	0	0	1	0	
sp|P43320|CRBB2_HUMAN	35	N	S	0	0	0	2	0	0	0	1	0	
sp|P43320|CRBB2_HUMAN	72	F	E	1	0	0	0	0	0	0	1	0	
sp|P43320|CRBB2_HUMAN	109	I	T	0	0	0	0	0	0	1	0	1	
sp|P43320|CRBB2_HUMAN	120	K	D	0	0	0	0	1	0	0	1	0	
sp|P43320|CRBB2_HUMAN	123	E	D	0	0	0	0	0	0	2	0	1	
sp|P43320|CRBB2_HUMAN	124	I	R	1	0	0	0	0	0	0	1	0	
sp|P43320|CRBB2_HUMAN	124	I	V	0	0	0	0	0	1	1	0	2	Cataract-specific
sp|P43320|CRBB2_HUMAN	125	I	V	0	0	0	0	0	1	0	0	1	
sp|P43320|CRBB2_HUMAN	147	Q	S	0	0	0	1	0	0	1	1	1	
sp|P43320|CRBB2_HUMAN	175	S	G	0	0	0	0	0	2	1	0	2	Cataract-specific
sp|P43320|CRBB2_HUMAN	180	P	M	0	0	0	0	0	0	2	0	1	
sp|P43320|CRBB2_HUMAN	181	H	Q	0	0	0	0	0	2	1	0	2	Cataract-specific
sp|P53672|CRBA2_HUMAN	124	V	K	0	0	0	0	0	1	0	0	1	
sp|P53673|CRBA4_HUMAN	22	F	E	1	0	0	0	0	0	0	1	0	
sp|P53673|CRBA4_HUMAN	36	V	F	0	0	2	0	0	0	0	1	0	
sp|P53673|CRBA4_HUMAN	59	H	C	0	0	1	0	0	0	0	1	0	
sp|P53673|CRBA4_HUMAN	106	R	Q	2	0	0	0	1	0	0	2	0	
sp|P53673|CRBA4_HUMAN	114	N	H	0	0	0	1	0	0	0	1	0	
sp|P53673|CRBA4_HUMAN	184	P	K	0	0	0	0	0	1	0	0	1	
sp|P53673|CRBA4_HUMAN	184	P	Q	0	0	0	0	0	1	2	0	2	Cataract-specific
sp|P53674|CRBB1_HUMAN	69	F	E	0	0	0	1	0	0	0	1	0	
sp|P53674|CRBB1_HUMAN	72	R	D	0	0	0	0	1	0	2	1	1	
sp|P53674|CRBB1_HUMAN	73	R	D	0	0	0	0	0	0	1	0	1	
sp|P53674|CRBB1_HUMAN	95	I	F	0	0	0	1	0	0	0	1	0	
sp|P53674|CRBB1_HUMAN	96	V	E	0	0	0	0	1	0	0	1	0	
sp|P53674|CRBB1_HUMAN	97	S	C	0	0	0	1	11	4	2	2	2	
sp|P53674|CRBB1_HUMAN	98	A	T	0	0	0	0	0	4	1	0	2	Cataract-specific
sp|P53674|CRBB1_HUMAN	113	M	IL	1	0	0	0	0	0	0	1	0	
sp|P53674|CRBB1_HUMAN	152	S	C	0	0	0	0	0	0	1	0	1	
sp|P53674|CRBB1_HUMAN	226	M	N	0	0	0	0	0	1	0	0	1	
sp|P53674|CRBB1_HUMAN	228	S	G	0	0	0	0	0	4	6	0	2	Cataract-specific
sp|P53674|CRBB1_HUMAN	230	R	H	0	0	0	0	16	0	0	1	0	
sp|P53674|CRBB1_HUMAN	231	R	H	0	0	0	0	3	0	0	1	0	
sp|P53674|CRBB1_HUMAN	234	D	M	0	0	1	0	0	0	0	1	0	
sp|P53674|CRBB1_HUMAN	236	Q	D	1	0	0	0	3	4	4	2	2	
sp|P53674|CRBB1_HUMAN	237	W	A	0	2	0	0	0	0	0	1	0	

Table S4. The list of possible mutations from nuclear cataract human lens samples. (a) Numbers of mutations observed in each crystallin. A total of 112 mutations were observed in our analyses. (b) Numbers of mutations observed in each sample.

a

Protein	Accession	#Mutations
Alpha-crystallin A chain	sp|P02489|CRYAA_HUMAN	15
Alpha-crystallin B chain	sp|P02511|CRYAB_HUMAN	12
Beta-crystallin A2	sp|P53672|CRBA2_HUMAN	1
Beta-crystallin A3	sp|P05813|CRBA1_HUMAN	4
Beta-crystallin A4	sp|P53673|CRBA4_HUMAN	7
Beta-crystallin B1	sp|P53674|CRBB1_HUMAN	16
Beta-crystallin B2	sp|P43320|CRBB2_HUMAN	13
Beta-crystallin B3	sp|P26998|CRBB3_HUMAN	3
Gamma-crystallin A	sp|P11844|CRGA_HUMAN	6
Gamma-crystallin B	sp|P07316|CRGB_HUMAN	8
Gamma-crystallin C	sp|P07315|CRGC_HUMAN	9
Gamma-crystallin D	sp|P07320|CRGD_HUMAN	5
Gamma-crystallin S	sp|P22914|CRYGS_HUMAN	13
Gamma-crystallin N	sp|Q8WXF5|CRGN_HUMAN	0

b

Sample	#Mutations
3-day	11
2-year	8
18-year	16
35-year	19
70-year	24
70-year-cataract	37
93-year-cataract	37
